# ECG-guided non-invasive estimation of pulmonary congestion in patients with heart failure

**DOI:** 10.1038/s41598-023-30900-9

**Published:** 2023-03-09

**Authors:** Aniruddh Raghu, Daphne Schlesinger, Eugene Pomerantsev, Srikanth Devireddy, Pinak Shah, Joseph Garasic, John Guttag, Collin M. Stultz

**Affiliations:** 1 Department of Electrical Engineering and Computer Science, Massachusetts Institute of Technology, Building 36-796, 77 Massachusetts Ave., Cambridge, MA 02139 USA; 2 Computer Science and Artificial Intelligence Laboratory, Massachusetts Institute of Technology, 32 Vassar St., Cambridge, MA 02139 USA; 3 Research Laboratory of Electronics, Massachusetts Institute of Technology, 77 Massachusetts Ave., Cambridge, MA 02139 USA; 4 Institute of Medical Engineering and Science, Massachusetts Institute of Technology, 77 Massachusetts Ave., Cambridge, MA 02139 USA; 5 Harvard Medical School, 25 Shattuck Street, Boston, MA 02115 USA; 6 Division of Cardiology, Massachusetts General Hospital, 55 Fruit St., Boston, MA 02114 USA; 7 Division of Cardiovascular Medicine, Brigham and Women’s Hospital, 75 Francis St., Boston, MA 02115 USA

**Keywords:** Cardiology, Biomedical engineering, Computer science

## Abstract

Quantifying hemodynamic severity in patients with heart failure (HF) is an integral part of clinical care. A key indicator of hemodynamic severity is the mean Pulmonary Capillary Wedge Pressure (mPCWP), which is ideally measured invasively. Accurate non-invasive estimates of the mPCWP in patients with heart failure would help identify individuals at the greatest risk of a HF exacerbation. We developed a deep learning model, HFNet, that uses the 12-lead electrocardiogram (ECG) together with age and sex to identify when the mPCWP > 18 mmHg in patients who have a prior diagnosis of HF. The model was developed using retrospective data from the Massachusetts General Hospital and evaluated on both an internal test set and an independent external validation set, from another institution. We developed an uncertainty score that identifies when model performance is likely to be poor, thereby helping clinicians gauge when to trust a given model prediction. HFNet AUROC for the task of estimating mPCWP > 18 mmHg was 0.8 $2\pm$ 0.01 and 0.$81\pm$ 0.01 on the internal and external datasets, respectively. The AUROC on predictions with the highest uncertainty are 0.50 $\pm$ 0.02 (internal) and 0.$56\pm$ 0.04 (external), while the AUROC on predictions with the lowest uncertainty were 0.86 ± 0.01 (internal) and 0.82 ± 0.01 (external). Using estimates of the prevalence of mPCWP > 18 mmHg in patients with reduced ventricular function, and a decision threshold corresponding to an 80% sensitivity, the calculated positive predictive value (PPV) is 0.$89\pm$ 0.01when the corresponding chest x-ray (CXR) is consistent with interstitial edema HF. When the CXR is not consistent with interstitial edema, the estimated PPV is 0.$78\pm$ 0.02, again at an 80% sensitivity threshold. HFNet can accurately predict elevated mPCWP in patients with HF using the 12-lead ECG and age/sex. The method also identifies cohorts in which the model is more/less likely to produce accurate outputs.

## Introduction

Heart failure (HF) remains a global public health burden with a prevalence of approximately 12% in individuals over 60 years of age^1^. Despite advances in diagnosis and therapy, patients with HF remain at increased risk for a variety of adverse outcomes including repeat hospitalization and death^2^.

The clinical evaluation of patients with HF involves an assessment of their underlying hemodynamics, with the cardiac output and mPCWP—an estimate of the left atrial pressure—being quantities that are used to assess the hemodynamic severity^3^. Although originally developed for patients who present with an acute myocardial infarction, the Forrester classification scheme has been validated in patients with heart failure and continues to provide prognostic information in these patients^4–7^. Forrester identified four distinct hemodynamic subsets based on two hemodynamic parameters: the mPCWP and the cardiac index (CI). Patients with a mPCWP $\le$ 18 and a CI $\ge$ 2.2L/min/m^2^ (“dry-warm”) had the best prognosis, while patients with a mPCWP > 18 mmHg and a CI < 2.2L/min/m^2^ (“wet-cold”) had the worst prognosis^4,8^. In addition, the mPCWP has particular importance in clinical assessment as a mPCWP > 18 mmHg is an independent predictor of adverse outcomes, while the CI is not^9,10^.

Unfortunately, both the mPCWP and CI are challenging to estimate from the clinical exam alone and are best are obtained via insertion of a pulmonary-artery catheter, which cannot always be performed safely and expeditiously in many clinical settings^11^. Current methods for the non-invasive estimation of mPCWP rely on analyses of mitral inflow velocities, obtained from a cardiac ultrasound^12–14^. However, this approach necessarily requires the acquisition of spectral Doppler images from an experienced sonographer and therefore may not be routinely available.

Deep learning (DL) holds the promise of leveraging non-invasive measurements that are easily obtained in a many clinical venues to estimate quantities that are typically acquired via an invasive study. Recent work developed DL models to estimate when the mPCWP is elevated using CXR images^15^. This approach, however, was not specifically developed for, nor tested in, patients with known HF. As ECG parameters have been shown to be correlated with abnormal hemodynamic profiles in some patient populations^16^, the 12-lead ECG serves as a potential data source that can be leveraged to estimate central pressures. Recently, we developed a deep learning model to estimate when the mPCWP is greater than 15 mmHg from ECG data using a heterogeneous cohort of patients who were referred for right heart catherization^17^. While the discriminatory ability of the model was good in a subset of patients who were referred for an evaluation of heart failure, a mPCWP cutoff of 15 mmHg at rest has not been shown to risk stratify patients with chronic heart failure^9^. Indeed, our previous model was intended to be a screening tool to rule out an elevated mPCWP in all patients over 60 years old^17^.

In the present study, we use an independent cohort of HF patients to develop and validate a model that uses the 12-lead ECG and simple demographic features (age, sex) to detect whether mPCWP > 18 mmHg. As in our previous work, our underlying hypothesis is that subtle changes in the ECG, which are difficult to detect by visual inspection alone, have diagnostic significance. We also developed a measure of model uncertainty using the predictive entropy of the model, which provides insight into when a patient-specific model prediction is likely to be untrustworthy.

## Results

### HFNet discriminatory performance

**Figure 1:**
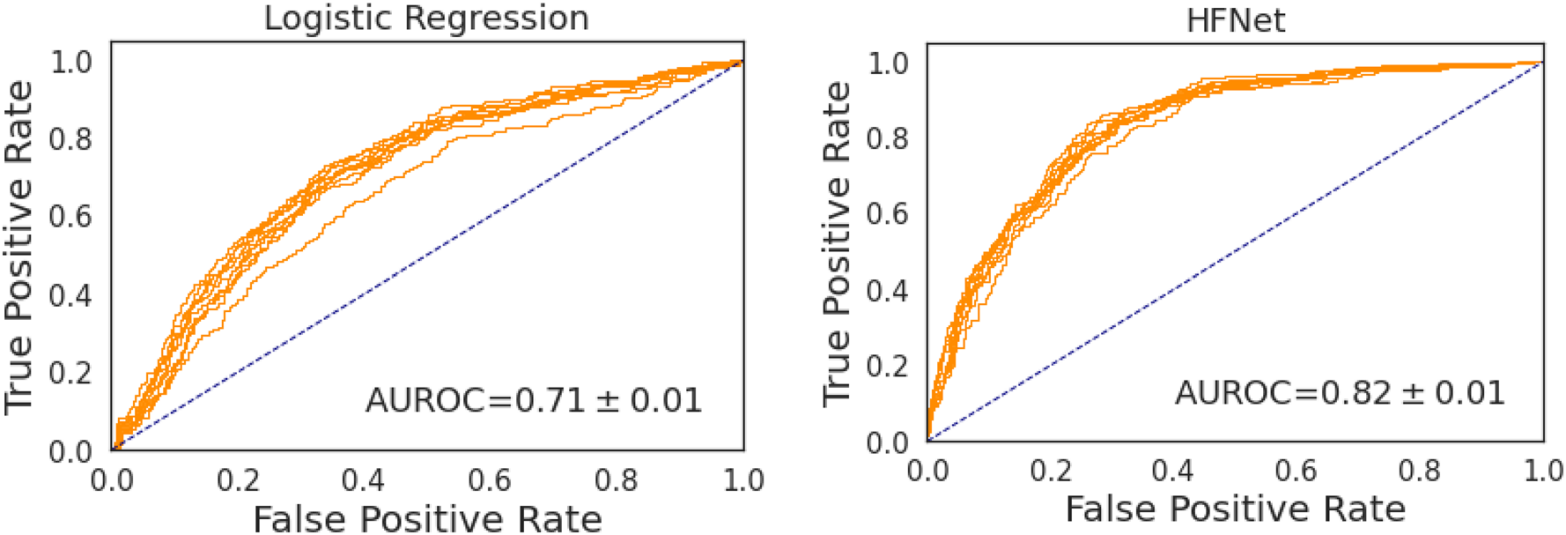
Receiver Operator Characteristic on the internal holdout set for (**a**) Logistic Regression baseline and (**b**) HFNet, showing 10 bootstraps. HFNet obtains significant improvements over the logistic regression baseline (paired t-test, *p* < 1e-10).

Table 1 shows the AUROC of our method, HFNet, and a baseline logistic regression model that uses ECG intervals, age, and sex to detect an elevated mPCWP > 18 mmHg. HFNet achieves an AUROC of 0.82 $\pm$ 0.01 on the internal holdout set, and significantly outperforms a logistic regression model trained using age/sex and ECG intervals. The associated receiver operator characteristic curves for each model are shown in Fig. 1.

**Table 1:** Dataset summary statistics.

Dataset	Institution	# RHCs	# patients	Age	% Female	% with mPCWP > 18 mmHg
Development	Hospital 1	5680	3014	63 + − 15	34%	37.7%
Internal Test	Hospital 1	1441	753	63 + − 15	38%	35.5%
External Validation	Hospital 2	2725	1249	56 + − 15	32%	34.3%

### A prediction-specific uncertainty score

**Figure 2:**
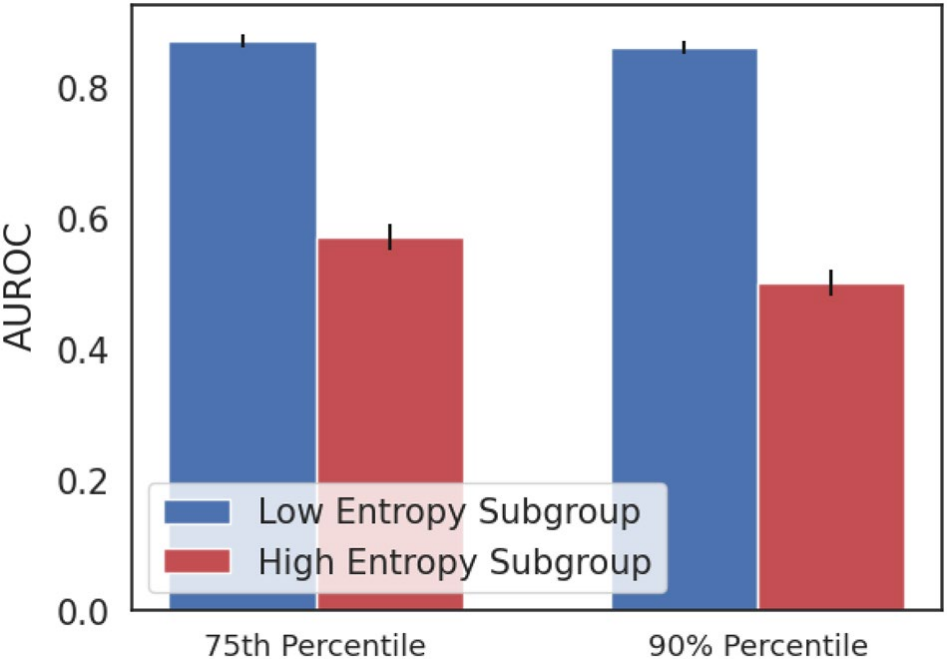
Performance stratified by entropy-based uncertainty score, showing mean and standard deviation over 10 bootstraps. HFNet performance is improved in cohorts with low uncertainty.

We developed a prediction-specific score, based on the prediction entropy, to identify instances in which model performance is likely to be poor. We hypothesized that the set of predictions with high prediction entropies correspond to a subset where model performance is poor. The discriminatory ability of the model is reduced in cohorts that have high predictive entropies (Fig. 2). We compute entropy percentiles on the “dev” set, and find that the cohort consisting of patients with entropies below the 90^th^ percentile has a testing set AUROC of 0.86 $\pm$ 0.01; on predictions within the top entropy decile, the AUROC is 0.50 +  − 0.02. At the 75th percentile entropy threshold, the AUROC is 0.87 $\pm$ 0.01, and within the top quartile of entropy values, the AUROC is 0.57 $\pm$ 0.02.

### HFNet predictive value

**Figure 3:**
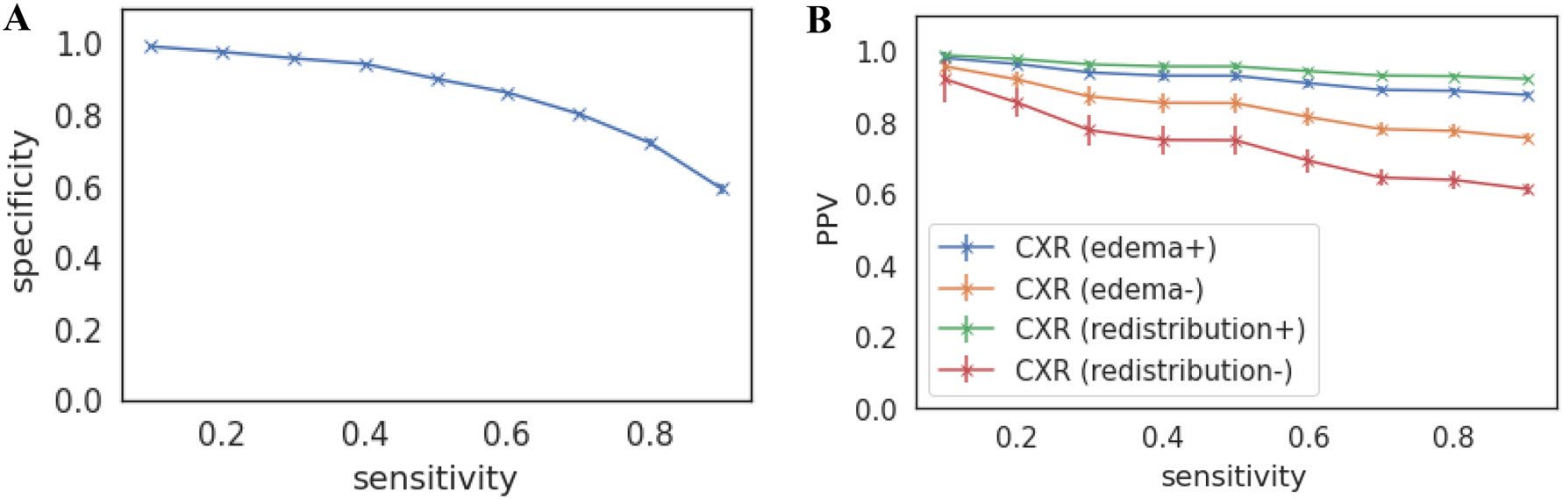
HFNet predictive value. (**A**) Specificity vs Sensitivity on the entire internal holdout set; (**B**) PPVs on the subcohort of the internal holdout set with reduced Ejection Fraction (EF < 40) and as a function of CXR findings on present on presentation. CXR(edema +) = evidence of interstitial edema; CXR(edema −) = interstitial edema absent; CXR(redistribution +) = evidence of pulmonary vascular redistribution; CXR(redistribution −) = no pulmonary vascular redistribution noted. Prevalence of different CXR findings in patients with reduced LV function taken from reference ^18^ . Plots show mean and standard deviation over 10 bootstraps.

Sensitivity and Specificity plots for HFNet for the entire cohort are shown in Fig. 3A. Using a threshold that achieves a sensitivity of 80% on the “dev” set, the associated specificity is 0.72 $\pm$ 0.01. Using our prediction-specific uncertainty score at the 90th percentile entropy threshold, and again an 80% sensitivity threshold, the model specificity increases to 0.83 $\pm$ 0.01. At the 75th percentile entropy threshold, the specificity increases to 0.91 $\pm$ 0.01.

To determine the predictive value of the model in the presence/absence of clinical findings consistent with volume overload, knowledge of the sensitivity, specificity and prevalence of an elevated mPCWP are needed (see Eq. 1). Since patients in our cohort do not reliably have a chest X-ray (CXR) in close proximity to when the RHC is performed, we rely on previously published data to estimate the prevalence of mPCWP > 18 mmHg in HF patients with reduced Ejection Fraction (HFrEF) in the setting of different CXR findings (see Supplementary Material)^18^. The sensitivity and specificity plots for patients with reduced LVEF are shown in the Supplementary Material. Figure 3B shows the calculated PPVs as function of sensitivity. In patients who have CXR findings consistent with interstitial edema, the PPV of the model is 0.$89\pm$ 0.01, using a threshold corresponding to an 80% sensitivity. In patients who do not have evidence of interstitial edema on CXR, the PPV of the model 0.$78\pm$ 0.02, again using a threshold that captures 80% of the true positive values. For the isolated CXR findings of vascular redistribution, the PPV of the model is 0.9 $3\pm$ 0.01, and when there are no signs of vascular redistribution the PPV is 0.$64\pm$ 0.03. Negative predictive values for the model are not as informative; i.e., in the presence of the CXR consistent with volume overload the NPV is 0.34 $\pm$ 0.02, and in the absence of such findings the NPV is 0.54 $\pm$ 0.03.

### HFNet tracks patient-specific changes in mPCWP

**Figure 4:**
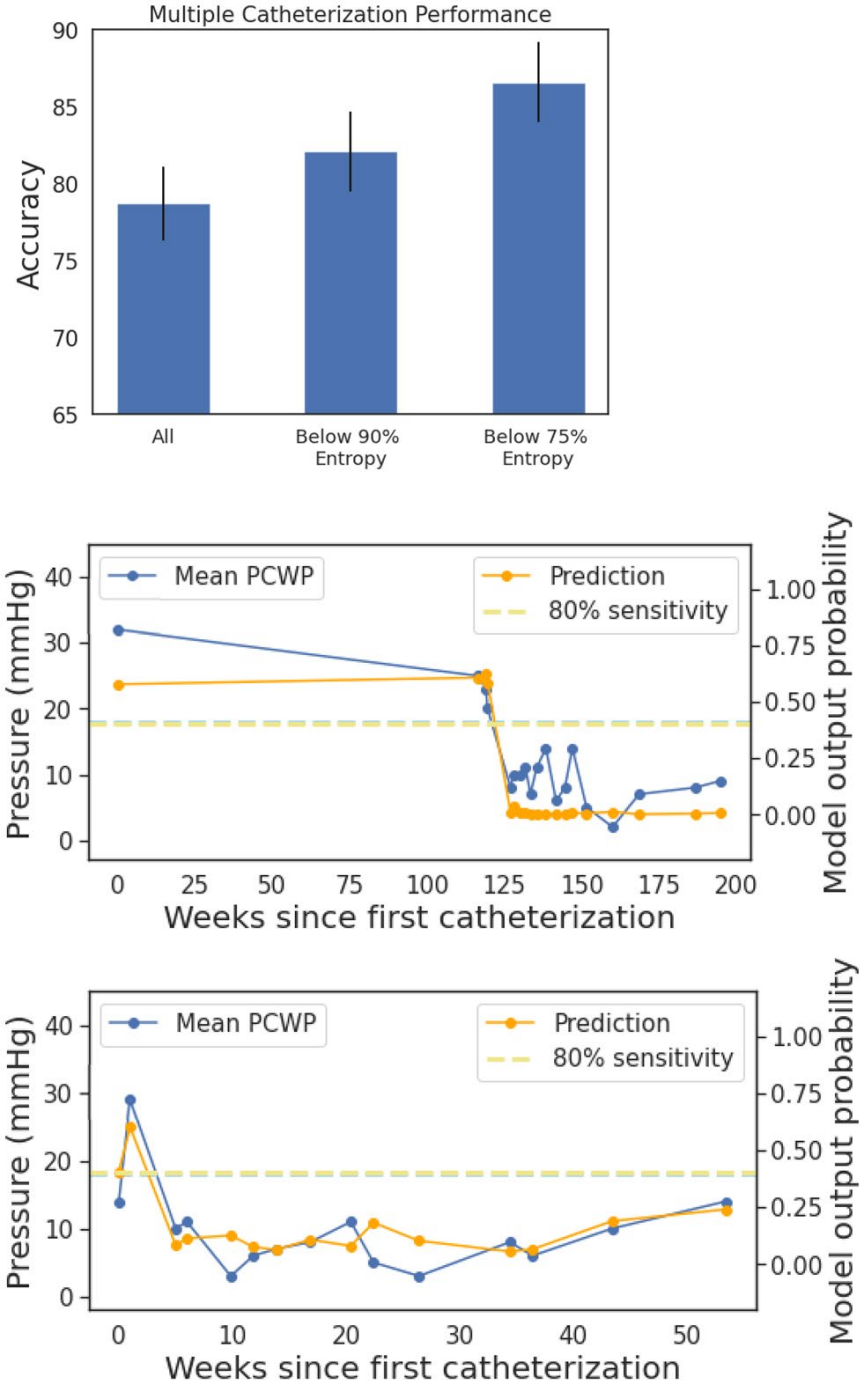
Performance on patients with multiple catheterizations. (**A**): Showing mean accuracy and standard error on sequences of catheterizations for patients at varying degrees of prediction uncertainty (N = 136). (**B**, **C**): Two examples of patients who had multiple catheterizations, showing measured mPCWP and model predictions. The blue dashed line corresponds to 18 mmHg mPCWP (left axis), and the dashed green line indicates the model output threshold corresponding to an 80% sensitivity level.

We assessed the ability of the HFNet to predict patient specific changes in hemodynamics. For each patient in the internal test set who had two or more right heart catheterizations, we computed the average accuracy of the model for detecting elevated mPCWP on that patient’s sequence of catheterizations; e.g., an accuracy of 100% means that the model reproduces the trend of mPCWPs measured across of that patient’s invasively measured mPCWPs. Figure 4A summarizes the results. For patients with multiple mPCWP measurements, the model achieves an average accuracy of 78.7 $\pm$ 2.4%, and the lower the entropy, the greater the accuracy. In the cohort with entropy values below the 75th percentile, the accuracy is approximately 87%.

Figure 4B also plots model predictions and mPCWP for two patients in the internal test set (additional examples are shown in the Supplementary Material). These data demonstrate that the model’s output can reproduce trends in the mPCWP over time and captures both reductions and increases in mPCWP over time.

### Saliency analysis and model sensitivity

**Figure 5:**
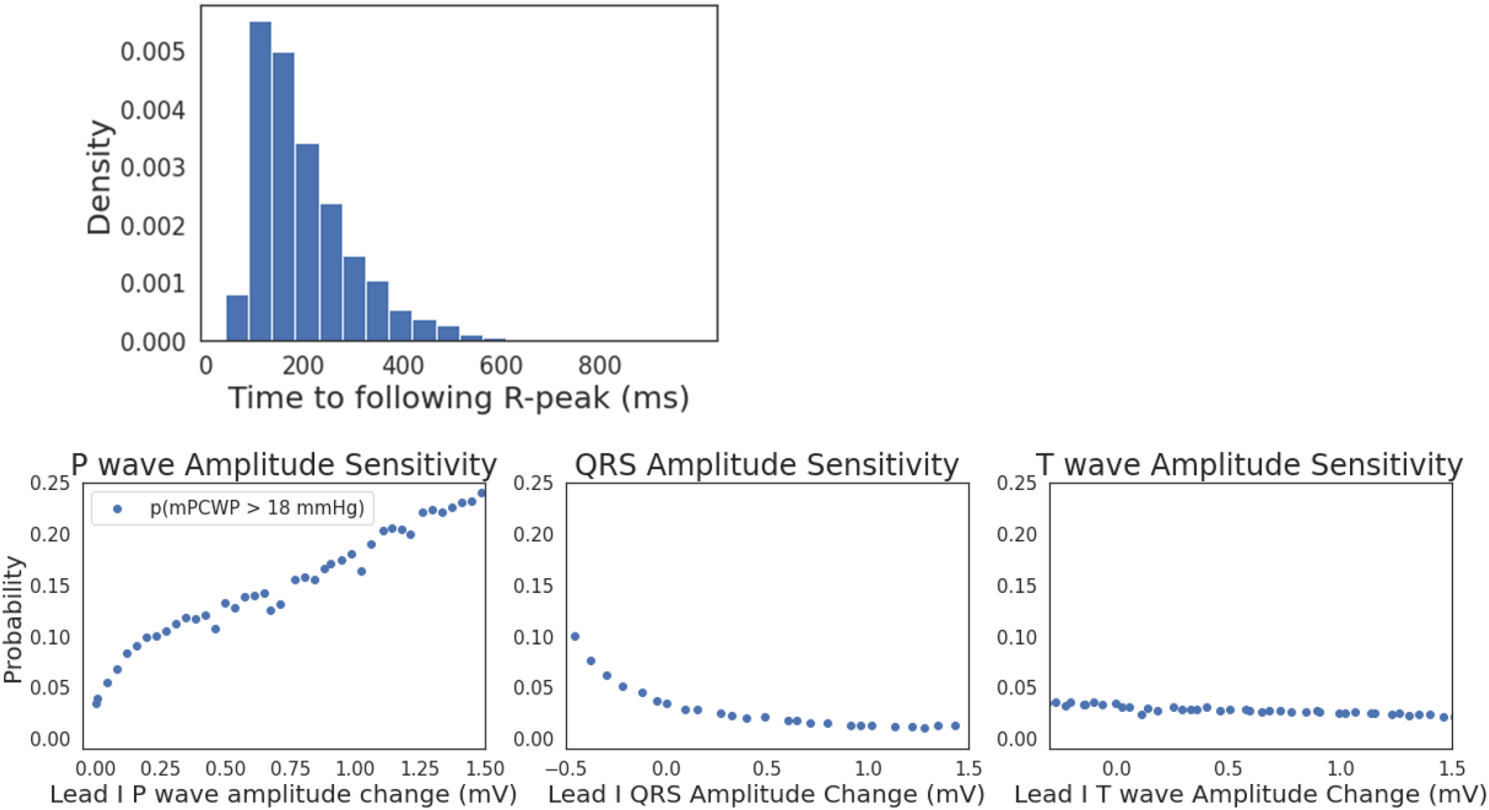
Model sensitivity and saliency map analysis. (**A**)**:** A saliency-map analysis showing that over 95% of the samples in the test set ECGs with the highest saliency score occur within 400 ms of the subsequent R-peak. (**B**)**:** Model output as a function of changes to the ECG, indicating that the model output is most sensitive to changes in the P-wave amplitude.

Saliency maps constitute one often-used method for identifying what portions of an ECG are most important for model decision making^17^. The method produces a “saliency value” for each sample in the ECG signal that quantifies the importance of that sample for the model’s prediction. For each ECG in the test set, we computed a saliency map for the input ECG, take the 100 highest saliency points in each ECG and compute the average time between the highest saliency point and the subsequent R-peak. Figure 5A presents the results. Over 95% of the highest saliency points are within 400 ms of the subsequent R-peak, indicating that the diastolic portion of the cardiac cycle has the greatest influence on model predictions.

To determine what specific ECG features (e.g., P-wave, QRS complex, T-wave) most influence model predictions, we generated synthetic ECG data using a previously described ECG generation tool that defines a dynamical system that can be used to produce synthetic 12-lead ECG waveforms^19^. The method enables us to systematically vary portions of the ECG signal to determine how sensitive the model output is to modifying the amplitude of different regions of the cardiac cycle. Results are shown in Fig. 5B. Of the three perturbations, increasing the P wave amplitude most affects the model output probability. Reducing the QRS amplitude elevates the predicted probability, though not as much as increased P wave amplitude. Changes to the T wave amplitude have little effect on the model output probability.

### Validation on an external dataset

**Figure 6:**
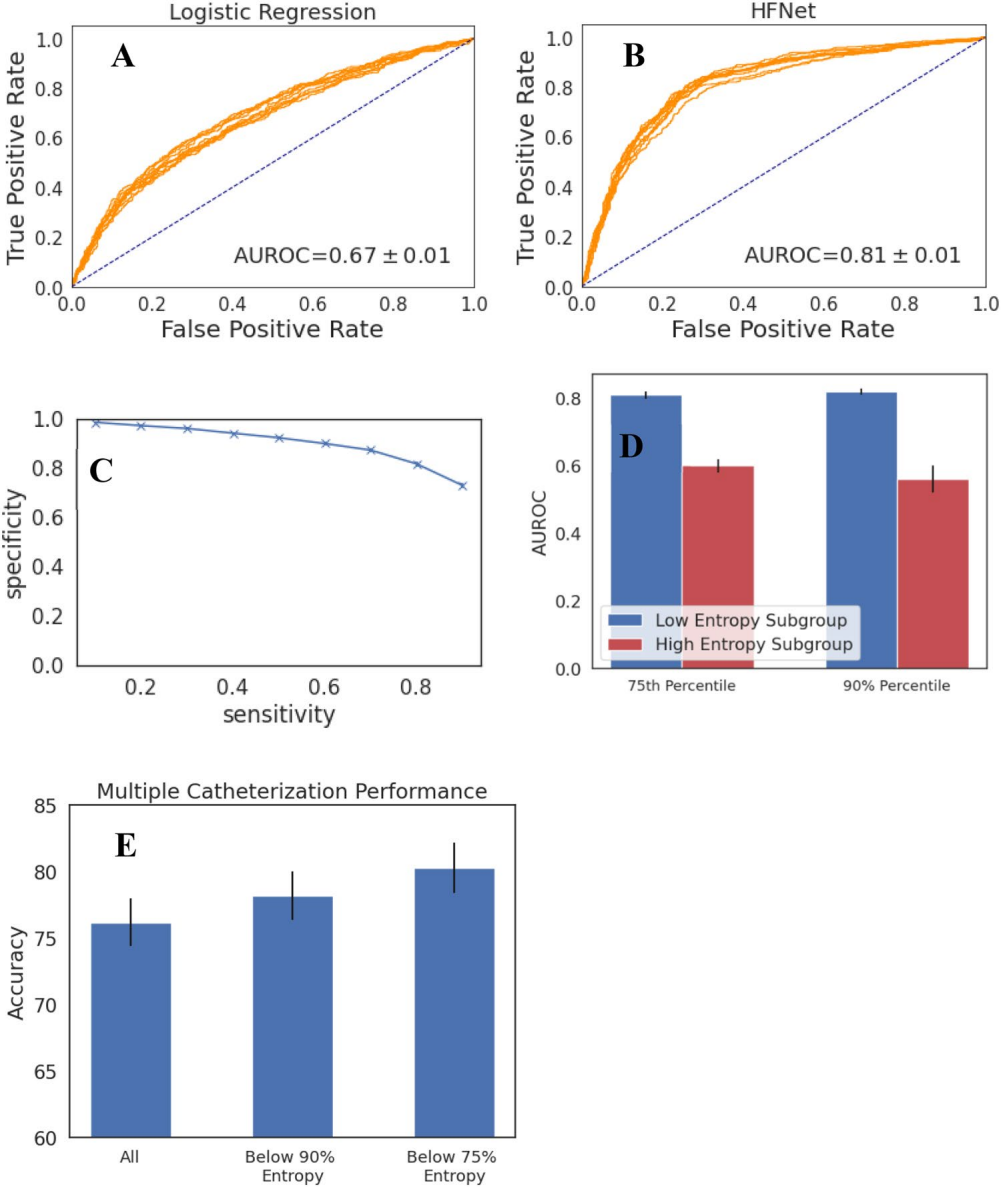
Validation on external dataset. On a dataset from a second institution, showing: (**a**),(**b**) Receiver Operator Characteristics for baseline logistic regression and HFNet over 10 bootstraps; (**c**) Performance stratified by prediction uncertainty score showing mean and standard deviation over 10 bootstraps; (**d**) Sensitivity and Specificity and PPV/NPV on the external dataset showing mean and standard deviation over 10 boostraps; (**e**) Sequence accuracy for patients with multiple catheterizations, showing mean and standard error (N = 281). The performance trends demonstrated on the internal test set are consistent in the external validation set.

We assessed HFNet performance using data from a second institution (See Table 1); Fig. 6 summarizes our key findings. HFNet significantly outperforms (*p* < 1e-10) the baseline logistic regression model on this dataset—HFNet achieves an AUROC of 0.$81\pm$ 0.01, compared to an AUROC of 0.67 $\pm$ 0.01 for the baseline model (Table 1, Fig. 6A, B).

Using our prediction-specific uncertainty score, we find that at the 90th percentile entropy threshold, the AUROC increases to 0.$82\pm$ 0.01, and on the subgroup with high uncertainty it is 0.$56\pm$ 0.04. Using the 75th percentile entropy threshold, the AUROC is 0.8 $1\pm$ 0.01, and in the cohort with high uncertainty, the AUROC is 0.60 $\pm$ 0.02. This suggests that the uncertainty score also is effective on the validation dataset (Fig. 6C).

Figure 6D shows the specificity vs sensitivity plot for the external holdout set, and it is similar to what was observed on our internal test set. Using a threshold that achieves a sensitivity of 80% on the dev set, the specificity on the external holdout set is 0.$82\pm$ 0.01; i.e., similar to what we obtained with the internal test set. Using our prediction-specific uncertainty score and the 90th percentile entropy threshold, at an 80% sensitivity threshold, the model specificity increases to 0.89 $\pm$ 0.01. At the 75th percentile entropy threshold, the specificity is 0.$92\pm$ 0.01. We do not have ejection fraction (EF) data for Hospital 2, so we could not calculate metrics in the reduced EF cohort with different clinical findings. Considering patients with multiple catheterizations, HFNet achieves an average accuracy of $76.2\pm$ 1.8% across multiple catherizations per patient. The accuracy increases to above 80% in the cohort for predictions that are in the lower 75th percentile of entropy.

## Discussion

In this study we developed a deep learning model to estimate the probability that a patient’s mPCWP is elevated, using a threshold of 18 mmHg in patients who have a prior diagnosis of HF. The resulting model has good discriminatory ability across a cohort of HF patients from two different hospitals—the AUROC scores on the internal and external holdout datasets were 0.8 $2\pm$ 0.01 and 0.81 $\pm$ 0.01 respectively.

While good discriminatory ability is a necessary condition for a clinical useful model, this, by itself, is far from sufficient^20^. For example, given that no model is ever, in practice, accurate 100% of the time, understanding failure modes of any model is important for high stakes decision making. We therefore developed a prediction-specific score, based on the entropy of the model output, which quantifies how certain the model is when it makes a prediction. We demonstrate that model discriminatory ability is significantly reduced when the associated entropy is high. Overall, the degree to which a specific model prediction should affect clinical decision making should be weighted by the associated prediction-specific reliability score.

When studying our model’s performance for patients with multiple catheterizations, we find that its predictions can approximately track the trends in the patient’s ground truth mPCWP measurements through catheterization. This is most evident when considering the model’s most confident predictions (with low entropy), and this trend is observed in both the internal and external datasets.

To gauge how HFNet could be integrated into clinical practice, we computed predictive values for patients with HF and reduced left ventricular ejection fraction (LVEF) in different clinical scenarios of interest. As the CXR forms a routine part of the evaluation of HF patients in the emergency room and in inpatients with a HF exacerbation, we focus on the added value of model predictions given different CXR findings. When signs of pulmonary congestion are evident on CXR, then the diagnosis of HF is very likely. In this setting HFNet provides incremental benefit, as its role would be to confirm the diagnosis of cardiogenic pulmonary edema. However, the CXR is not always a reliable indicator of cardiogenic pulmonary edema in patients with HF. For example, several studies suggest that approximately 20% of patients with acute decompensated HF present with normal CXRs and up to 50% of patients with HF have a mPCWP > 18 mmHg and CXR findings that are not consistent with vascular redistribution or interstitial edema^18,21^. Hence, arriving at the right diagnosis is challenging in HF patients with suspected decompensated heart failure who have unremarkable chest x-ray findings. Plasma levels of B-type natriuretic peptide (BNP) and N-Terminal pro-BNP (NT-proBNP) have high negative predictive value for ruling out acute heart failure in patients who present with dyspnea^22^. However, since BNP and NT-proBNP levels can be persistently elevated in patients with chronic HF, the positive predictive value of natriuretic peptides in the diagnosis of acute decompensated HF in these patients is not as clear^23^.

Using estimates of the prevalence of mPCWP > 18 mmHg in HFrEF patients, given different radiographic pulmonary findings, and a decision threshold corresponding to a sensitivity of 80%, we estimate that the PPV is 0.$89\pm$ 0.01 when the CXR is consistent with interstitial edema. When signs of interstitial edema are absent the PPV is 0.$78\pm$ 0.02, again at a sensitivity of 80%. HFNet can therefore provide information that complements CXR findings in patients with HF. Indeed, an HFNet prediction consistent with an elevated mPCWP is meaningful even if the CXR does not show signs of pulmonary edema; i.e., a diagnosis of acute HF is likely in patients with a positive HFNet prediction and negative CXR findings. Moreover, given that a persistently elevated mPCWP after directed therapy for a HF is associated with adverse outcomes^24^, HFNet could be part of the evaluation of HF patients before discharge, even when the CXR may be unremarkable. When considering whether a given inpatient with HF is ready for hospital discharge, a positive prediction should raise the suspicion that their filling pressures remain elevated and that additional inpatient therapy is required. Lastly, we note that while we focus on a decision threshold corresponding to a sensitivity of 80%—a common cutoff used in the medical literature—we note that higher PPVs are achieved at lower sensitivity values (as shown in Fig. 3).

Deep learning models suffer from the criticism that they are opaque algorithms because it is challenging to understand what these models have learned in practice^20^. In order to probe what HFNet has learned, we used two complementary approaches. The first method, saliency map analysis, is a widely used approach for understanding what portions of the input data have the greatest influence on model predictions. The second approach used synthetic ECG sequences to systematically vary the amplitude of different portions of the ECG to study how the model output varies as different portions of the ECG are modified. Both approaches suggest that HFNet preferentially focuses on the diastolic portion of the cardiac cycle, with the amplitude of the P-wave having the greatest influence on model output. These data are consistent with the longstanding clinical intuition that the mPCWP is a reasonable estimate for left sided pressures at end diastole in many patients^25^. While this does not constitute a comprehensive explanation of what the model has learned, it does suggest that HFNet has garnered information that is consistent with our prior understanding of human pathophysiology.

While we believe these data suggest that HFNet has a role in the evaluation and management of patients with HF, our study has limitations. Our approach estimates whether the mPCWP is above a threshold or below it, rather than predicting the precise value of the mPCWP itself. Although a finer grained prediction would be more useful, the method here identifies patients who have pulmonary congestion and helps to prioritize who should have additional investigative studies. More data are needed to develop a robust method that can precisely estimate the mPCWP. Also, when curating our dataset of paired ECGs and mPCWP measurements, we ensured that the ECG and catheterization were performed on the same day, however, the specific time of catheterization relative to the ECG recording is not known and this introduces some uncertainty in our results. Obtaining the ECG immediately prior to catheterization could improve predictive performance. In addition, our ECG dataset was not restricted to patients with normal sinus rhythm—as we hope to develop a method that would work in the setting of different cardiac arrythmias. However, assessing the performance in cohorts with significant arrhythmias are challenging as our set of ECGs with significant arrhythmias is small; e.g., less than 10% of our ECGs have atrial fibrillation. Additional work is needed to fully assess the performance of our model on patients with significant arrhythmias. Furthermore, our estimates for the PPV and NPV rely on estimates of the prevalence of elevated mPCWP that were derived from prior studies of patients with HF and reduced LVEF. Since we have limited data on the demographics of this population (i.e., only age, sex, filling pressures, LVEF), it is difficult to make definitive statements about how these results generalize to other populations that may have very different characteristics. Further prospective studies are needed to verify that these estimates are applicable to the wide swath of patients that are seen in modern day practice; for example, to evaluate how the model performs on patients who had an ECG but who would not have undergone catheterization as part of their care.

Overall, our study suggests that there is a role for deep learning models in the estimation of central cardiac pressures in patients with heart failure. The method has the potential to provide clinically useful information when invasive assessment of central hemodynamics is either not possible or feasible.

## Methods

### Datasets

We develop and validate our model using datasets from two different hospitals. Our first dataset consists of 7121 records from 3767 unique patients who underwent cardiac catheterization at Massachusetts General Hospital (Hospital 1). All patients had a diagnosis of HF (according to ICD 9/10 codes in their medical record) within the 1 year prior to their catheterization date.

This dataset is split into an 80% development set, used to train predictive models, and a 20% internal holdout test set, used for model evaluation. Datasets are constructed such that no data from a single patient appears in different data splits; i.e., all data splits are done on a per-patient basis. We further split the development set on a per-patient level using an 80–20 split into training and “dev” sets. The training set is used to train the model and the dev set is used to determine when training is completed.

Our second dataset consists of 2725 records from 1249 unique patients who underwent cardiac catheterization at the Brigham and Women’s Hospital (Hospital 2). As with data from MGH, these patients all had a diagnosis of heart failure (according to ICD 9/10 codes in their medical record) within the 1 year prior to their catheterization date. We used this entire dataset as an external validation set for model evaluation.

Each record in the datasets consists of: the mean Pulmonary Capillary Wedge Pressure (as measured by cardiac catheterization), a 10-s, 12-lead ECG recorded by the same system (GE Healthcare MUSE) on the same day as the catheterization procedure, and basic demographic information (age/sex). Dataset details are summarized in Table 2.

**Table 2:** Model performance (AUROC) on test data. HFNet significantly outperforms the baseline logistic regression (LR) model.

Model	AUROC	
	Internal test set	External holdout set
LR	0.71 + − 0.01	0.67 + − 0.01
HFNet	**0.82 + − 0.01** *	**0.81 + − 0.01** *

Significant values are in bold.

Key: *: *p* value < 1e − 10.

### Data pre-processing

We resampled all 12-lead ECGs at 250 Hz (the raw ECG signals were recorded at either 250 Hz or 500 Hz) and removed any ECGs containing non-physical voltage values (> 5 mV in magnitude) in any lead. We additionally removed ECGs that had any leads that were zero-valued for more than 5 s. We normalized the continuous age feature using Z-scoring based on the mean and standard deviation of age in the training set. We binarized the sex feature and then subtracted 0.5 to center it on zero; i.e., 0.5 denotes male and − 0.5 denotes female. The continuous mPCWP measurements were binarized using the threshold of 18 mmHg.

### Baseline model

We constructed a Logistic Ridge Regression model to predict elevated mPCWP from age, sex, and intervals extracted from the ECG (heart rate and PR, QRS, and QT intervals) to predict whether the mPCWP is above 18 mmHg. The regularization strength was chosen to be the value that gave the best AUROC on the dev set. The intervals were normalized using z-scoring based on training set statistics, and the age and sex features were normalized as described above.

### Model development

We developed a deep learning model, HFNet, which estimates whether a patient’s mPCWP is over 18 mmHg, using the 10-s 12-lead ECG, age, and sex as input features. We included age and sex in the model since we hypothesized that that these features encode some prior information about filling pressures; e.g., older age and male sex are associated with a higher prevalence of cardiac disease. The model combines a convolutional encoder for the ECG and a fully connected network encoder for the demographic features. The model’s weights and biases are initialized as previously described^17^. The model is then trained to minimize the binary cross-entropy loss between its output, corresponding to mPCWP > 18 mmHg, and the binary label of whether the pressure measured during catheterization was elevated or not. Training proceeds for at most 50 epochs using the Adam optimizer with a learning rate of 1e-3, however, we use early stopping based on the dev set AUROC score to prevent model overfitting. Full architectural details of the model are in the Supplementary Material.

### Statistics

We obtain measures of uncertainty in performance using empirical bootstrapping; we sample data points at random, with replacement, obtaining 10 bootstrapped sets that have the same size as the original set. Performance metrics are computed on each of the bootstraps, and then the mean/standard deviation of performance across these bootstraps is reported. Paired t-tests were used to calculate *p*-values to assess statistical significance.

Positive and Negative Predictive Values as a function of sensitivity, specificity, and prevalence were computed using the following relationships:

1 $$\begin{gathered} PPV = \frac{sensitivity*prevalence}{{sensitivity*prevalence + \left( {1 - specificity} \right)*\left( {1 - prevalence} \right)}} \hfill \\ NPV = \frac{{specificity*\left( {1 - prevalence} \right)}}{{specificity*\left( {1 - prevalence} \right) + \left( {1 - sensitivity} \right)*prevalence}} \hfill \\ \end{gathered}$$

### Identifying untrustworthy predictions

Assessing whether a given prediction made by the model is trustworthy or not is important for practical use, since it helps clinicians decide in which cases to trust a given model prediction. We hypothesized that we could use the predictive entropy of the model’s output as a way of determining whether the model’s output is trustworthy or not on a per-ECG basis. Denoting the model input to be $x$, and the model output to be $\widehat{y}$ (representing the probability that mPCWP > 18 mmHg), the predictive entropy is defined as:

$$Entropy\left( x \right) = { } - \left( {\hat{y}\left( x \right){ }\log \hat{y} + \left( {1 - \hat{y}\left( x \right)} \right)\log \left( {1 - \hat{y}\left( x \right)} \right)} \right)$$

Here, $x$ denotes the ECG and age, sex (inputs to the model); and $\widehat{y}(x)$ is the model output (the prediction). We hypothesize that large entropy values (which correspond to the predicted probability being near 0.5) arise in situations where the model is least reliable, and the lowest entropy values (which correspond probabilities near 0 or 1) arise for inputs where the model’s prediction is most reliable.

### Role of funders

This work was funded by a competitive grant from Quanta Computers Inc. that was awarded to CMS. The funder was not involved in any aspect of data collection, algorithm design, analysis of results, or writing the paper. The funder did not review the manuscript prior to publication.

### Ethical approval

Approval for this retrospective study was obtained via the Mass General Brigham Institutional Review Board, Protocol number: 2020P000132. The IRB waived the need for informed consents from participants. All methods were performed in accordance with the relevant guidelines and regulations.

## Supplementary Information

Supplementary Information.

## Data Availability

HFNet is available for general use at https://github.com/aniruddhraghu/hfnet**.** Additional information related to this study is available on reasonable request to the corresponding author. All requests should be directed by email to the corresponding author. De-identified data may be available, with IRB approval.
