# Supplementary material for: ECG-guided non-invasive estimation of pulmonary congestion in patients with heart failure
Source: Sci Rep. 2023 Mar 9;13:3923. doi: 10.1038/s41598-023-30900-9 (PMC9998622; doi:10.1038/s41598-023-30900-9)
Supplement: Supplementary file 1 — Supplementary Information. [file 41598_2023_30900_MOESM1_ESM.docx]

**Supplementary Material for ECG-guided Non-invasive Estimation of Pulmonary Congestion in Patients with Heart Failure**

**Table of Contents**

| Model Architecture | 2 |
| --- | --- |
| Prevalence of mPCWP>18mmHg as a function of different CXR findings | 4 |
| PPV and NPV as a function of sensitivity, specificity and prevalence | 5 |
| Patient characteristics for cohort with reduced LVEF | 6 |
| Sensitivity and Specificity for cohort with reduced LVEF | 7 |
| HFNet tracks changes in mPCWP: examples from the internal test set. | 8 |
| HFNet tracks changes in mPCWP: examples from the external validation set. | 9 |
| References | 10 |

Model Architecture

Our model has three components: an ECG encoder, a demographic features encoder, and a classifier.


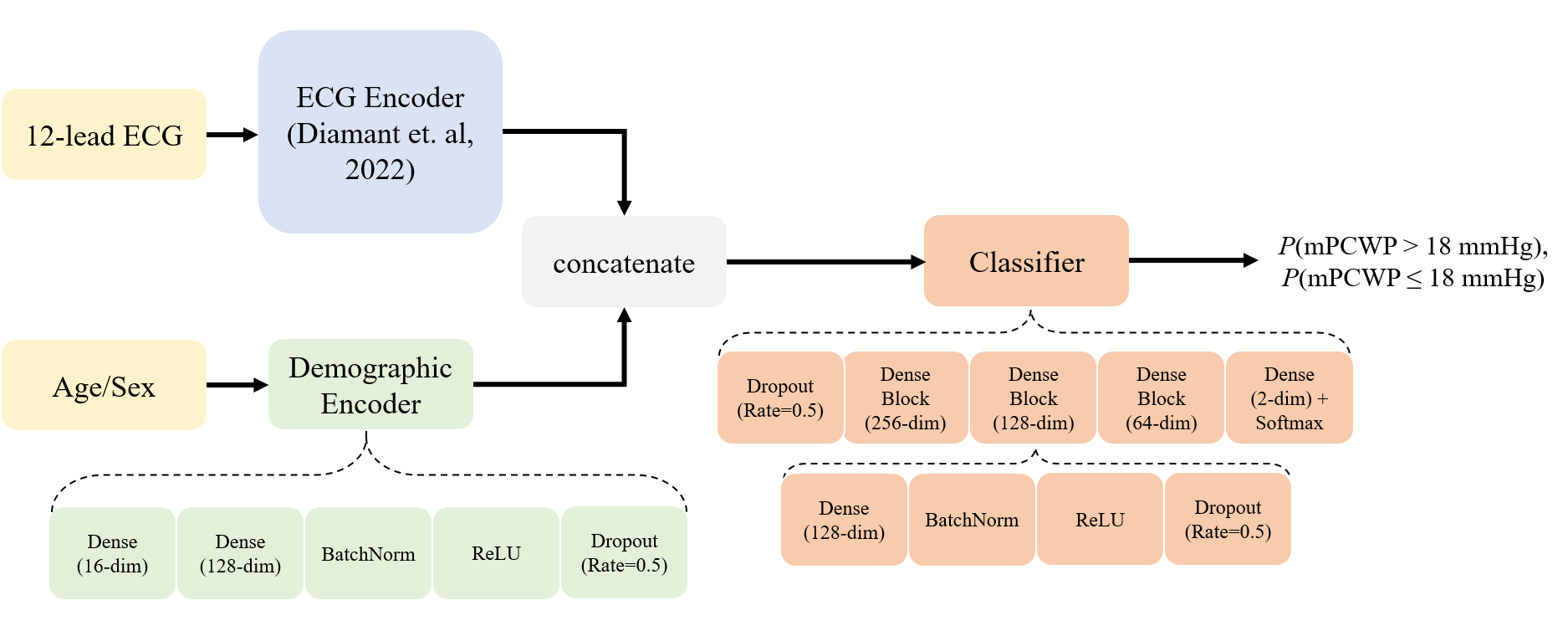


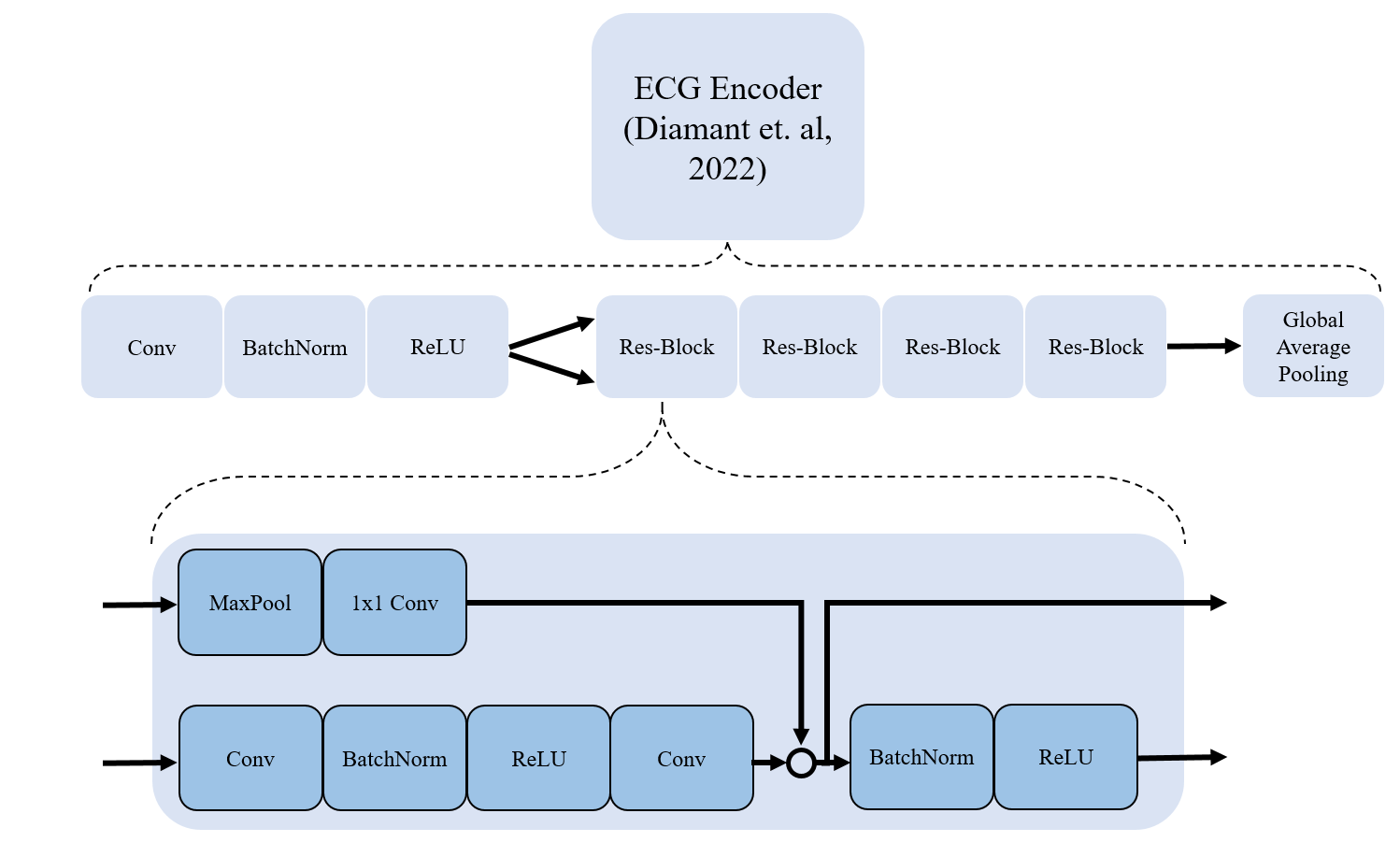


ECG encoder: Our ECG encoder is a convolutional neural network (CNN). We adopt the encoder architecture from Diamant et al for the encoder. This model consists of a residual architecture followed by a Global Average Pooling layer to produce a 320-dimensional representation of the 12-lead ECG signal.

Demographic features encoder: The demographic features encoder is a fully connected neural network. The two-dimensional vector with normalized age and sex is first projected into a 16-dimensional space with a Dense layer. This is then passed through a fully connected block with a Dense layer mapping into 128-dimensions, followed by Batch Normalization, ReLU activation and Dropout (rate 0.5).

Classifier: The classifier first concatenates the output from the ECG encoder and demographic features encoder, yielding a 448-dimensional vector, and then applies Dropout (rate 0.5). Then, 3 blocks of Dense, BatchNorm, ReLU, Dropout (rate 0.5) are applied, with Dense layer output dimensionalities being 256, 128, and 64 dimensions. This is followed by a final Dense layer with 2 dimensional output, which corresponds to the probability of mPCWP being above 18 mmHg and below 18 mmHg respectively.

**Table 1:** Prevalence of mPCWP>18mmHg as a function of different CXR findings. Taken from reference 1

| CXR Finding | Prevalence of mPCWP>18mmHg in patients with HFrEF | Sensitivity of positive CXR for detecting mPCWP>18mmHg |
| --- | --- | --- |
| Interstitial edema | 0.83 | 27% |
| No interstitial | 0.68 | - |
| Pulmonary Vascular Redistribution | 0.89 | 65% |
| No Pulmonary Vascular Distribution | 0.52 | - |

**Table 2**: Characteristics of patients with reduced ejection fraction (EF)

| Dataset | # RHCs | # patients | Age | % Female | % with  mPCWP > 18mmHg | Mean and standard deviation of EF |
| --- | --- | --- | --- | --- | --- | --- |
| Development | 1535 | 1053 | 63+- 15 | 25% | 59.2% | 24 +- 8 |
| Internal Test | 358 | 245 | 64 +-14 | 30% | 62.2% | 24 +- 8 |


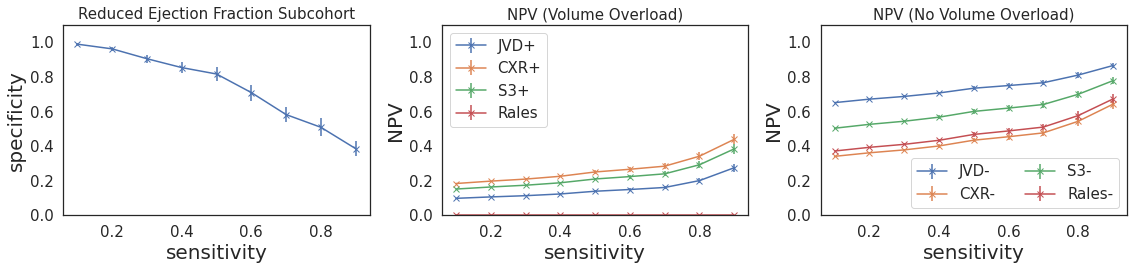


Figure 1: Sensitivity and Specificity for cohort with reduced LVEF, showing mean and standard deviation over 10 bootstraps.


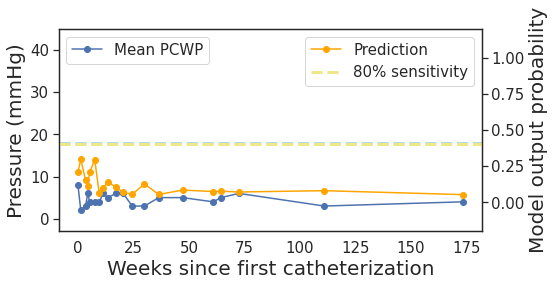

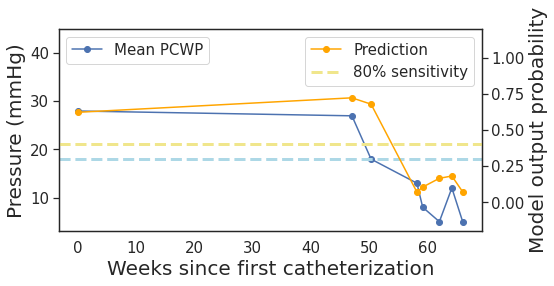

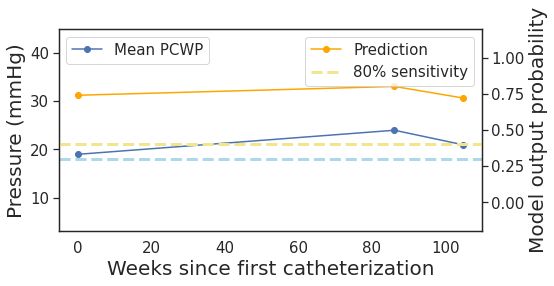

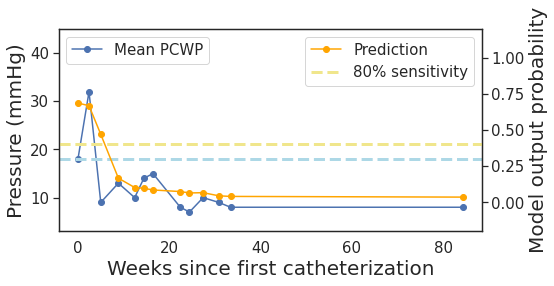

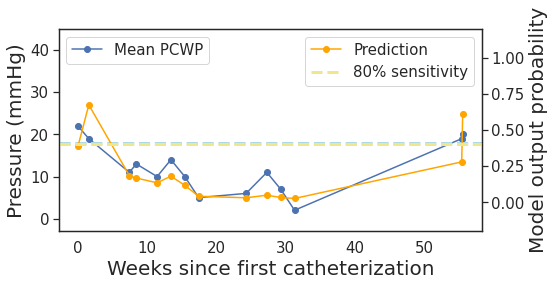

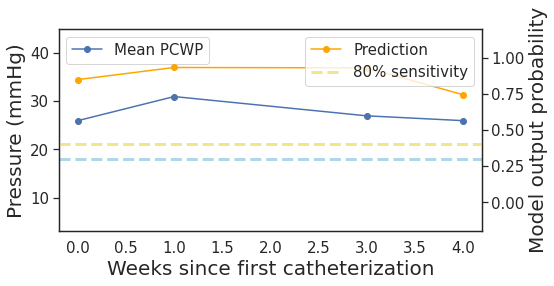

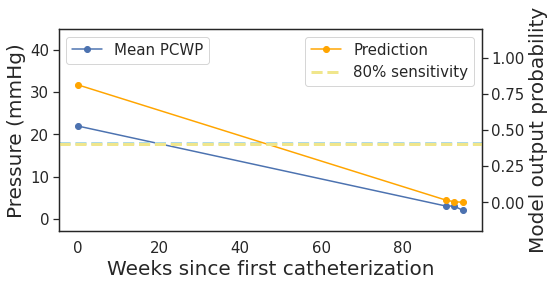

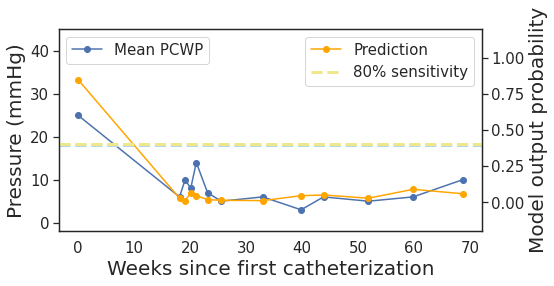

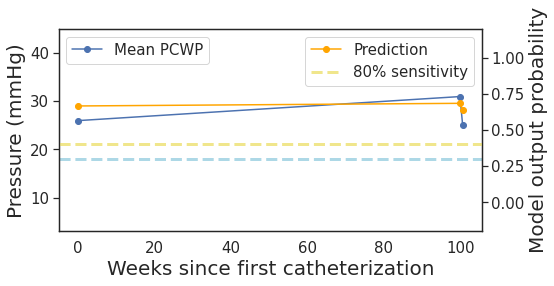

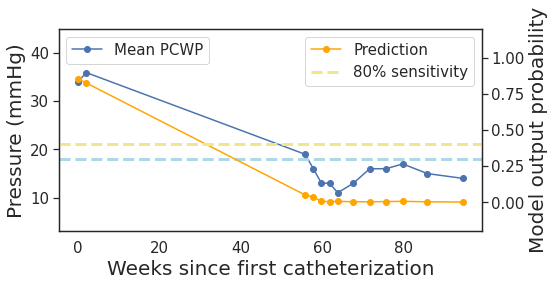


Figure 2: Multiple catheterization: further examples from the internal test set. The blue dashed line corresponds to 18mmHg mPCWP (left axis), and the dashed green line indicates the model output threshold corresponding to an 80% sensitivity level.


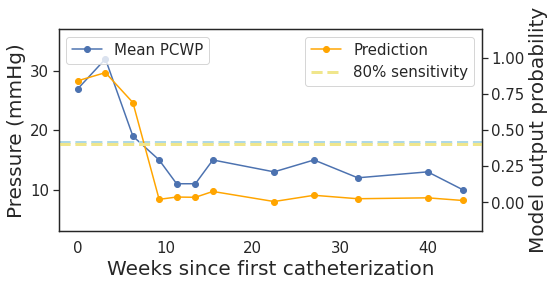

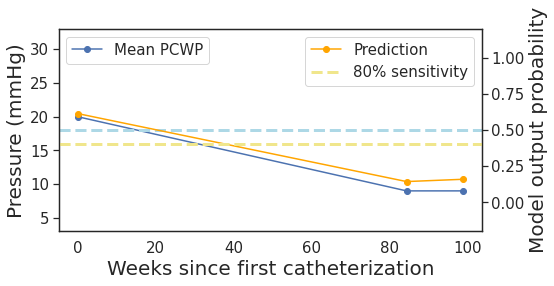

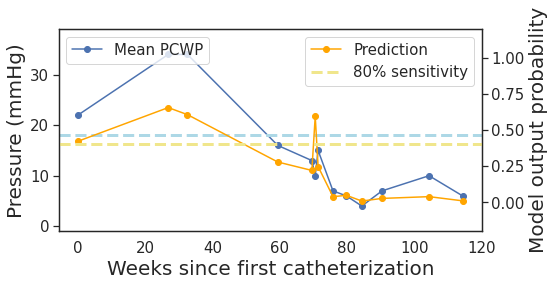

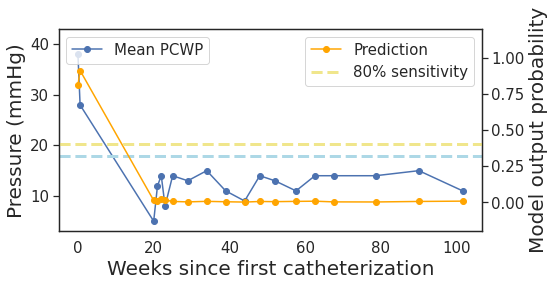

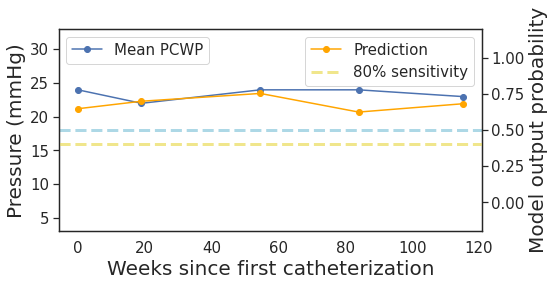

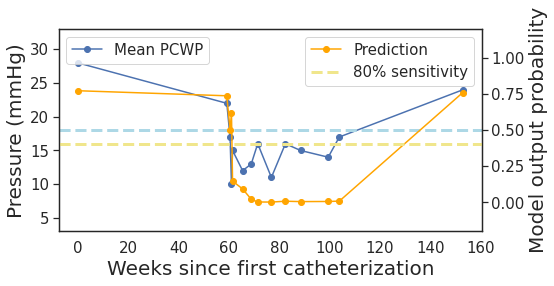

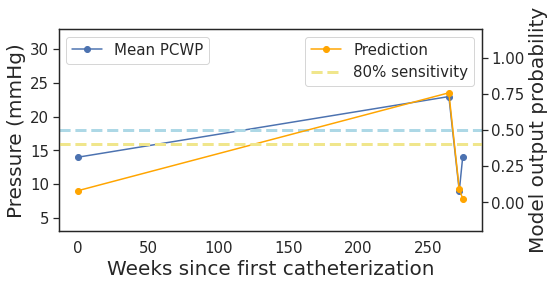

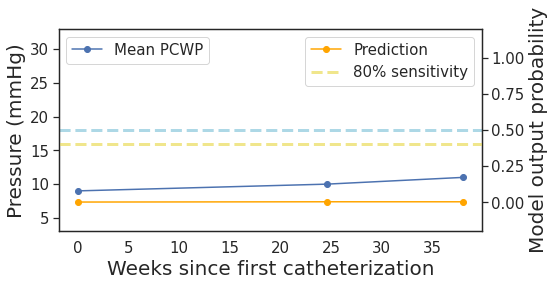

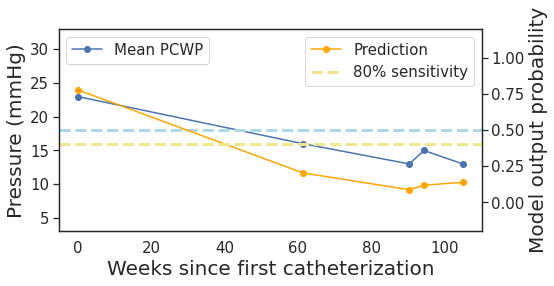

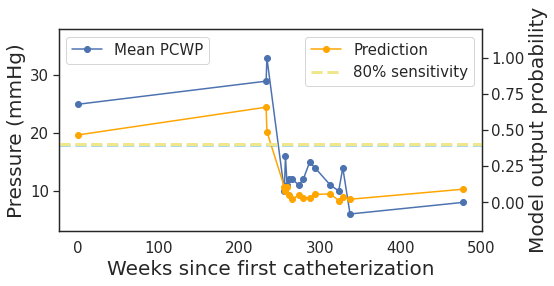


Figure 3: Multiple catheterization: further examples from the external validation set. The blue dashed line corresponds to 18mmHg mPCWP (left axis), and the dashed green line indicates the model output threshold corresponding to an 80% sensitivity level.

**References**

1. Butman SM, Ewy GA, Standen JR, Kern KB, Hahn E. Bedside cardiovascular examination in patients with severe chronic heart failure: importance of rest or inducible jugular venous distension. *J Am Coll Cardiol* 1993; **22**(4): 968-74.
